# Evidence of diversification and geographic separation in a lineage of Campylobacter jejuni co-circulating in cattle and humans

**DOI:** 10.1099/mgen.0.001553

**Published:** 2025-10-31

**Authors:** Jose A. Rodrigues, Karla A. Vasco, Heather M. Blankenship, Wonhee Cha, Sanjana Mukherjee, Rebekah E. Sloup, Samantha L. Carbonell, Marty K. Soehnlen, Shannon D. Manning

**Affiliations:** 1 Department of Microbiology, Genetics, and Immunology, Michigan State University, East Lansing, Michigan, USA; 2 Michigan Department of Health and Human Services, Bureau of Laboratories, Lansing, Michigan, USA

**Keywords:** *Campylobacter jejuni*, epidemiology, evolution, genomics, pangenome

## Abstract

*Campylobacter jejuni*, a leading cause of gastroenteritis, is acquired by consuming contaminated food products or via direct contact with animal reservoirs like cattle and poultry. Prior studies have identified specific *C. jejuni* lineages such as sequence type (ST)-982 and ST-61, to be associated with cattle, while others are linked to chickens and other animal sources. Since cattle-associated lineages were more commonly resistant to tetracycline and detected in patients with a history of cattle contact, we performed comparative genomics on 61 cattle- and 175 human-derived strains collected during an overlapping time period in Michigan. Pangenome analyses and a core-gene phylogeny revealed a high degree of genomic similarity among cattle-associated lineages regardless of strain source, while a machine learning approach predicted that the ST-982 human strains originated from cattle. Further characterization of these closely related strains using high-quality SNP clustering demonstrated that the human and cattle strains differed by >260 SNPs. The cattle genomes also have less unique genes with fewer accessory and virulence genes than the human genomes, providing evidence for diversification within and adaptation to the cattle host. Comparing the core-gene MLST (cgMLST) profiles to those from 723 published ST-982 genomes identified multiple sequence clusters that varied in frequency by US region. Together, these data illustrate that genetically similar ST-982 strains are co-circulating in cattle and humans. They also suggest that clonal expansion of cattle-associated lineages may contribute to regional differences in genotype distributions and antibiotic resistance frequencies, which likely occur due to varying selective pressures present in each region.

**Impact Statement**

*Campylobacter jejuni* is a genetically diverse foodborne pathogen and the leading cause of gastroenteritis. Herein, we used whole-genome sequencing to compare human clinical (*n*=175) and cattle-derived (*n*=61) *C. jejuni* strains collected in Michigan during the same time frame. We describe the genomic epidemiology of cattle-associated sequence types (STs), such as ST-982, at a local and national level and demonstrate that the human ST-982 strains are highly related to cattle strains from different herds. A machine learning algorithm was applied and predicted the source of all human-derived ST-982 strains to be cattle. A comparison of these genomes to 723 published ST-982 genomes demonstrated that this lineage is genetically diverse with evidence of diversification in different geographic locations in the USA. Understanding the evolution and epidemiology of a prevalent *C. jejuni* lineage, which is circulating in cattle and causes high frequencies of clinical infections in the same area, could aid in targeted prevention and mitigation efforts during outbreak and case investigations involving cattle and other important reservoirs.

## Data Summary

Whole-genome sequencing data were uploaded to the National Center for Biotechnology Information (NCBI) within BioProjects PRJNA1023301 and are listed in Table S1 with metadata and strain numbers. Genome sequences are available in NCBI BioProjects PRJNA305281, PRJNA368990 and PRJNA951423. Bioinformatic scripts are available at https://github.com/RodriguesJA/Cjejuni_Cattle_MI. The .nwk file and ST-982 metadata can be viewed through https://auspice.us/. All authors confirm support of data, codes and protocols.

## Introduction

*Campylobacter* spp. are the most common cause of human bacterial gastroenteritis in the USA, affecting up to 1.5 million people annually [1]. They have a broad host range, and food animals such as poultry, cattle and pigs serve as reservoirs [2]. In the USA, campylobacteriosis is often associated with traveller’s diarrhoea due to consumption of contaminated food products or exposure to contaminated environmental or food animal reservoirs [3].

Although poultry consumption is considered the main food-associated risk factor for campylobacteriosis [4], cattle products have emerged as an important source [3,5, 6]. Indeed, dairy products like unpasteurized milk have been linked to an increasing number of outbreak-associated cases [7,8], and attribution studies have identified cattle as the source of 29–68% of campylobacteriosis cases depending on the geographic location [4,6,9]. Consistent with findings, we previously showed that campylobacteriosis cases from Michigan were significantly more likely to report recent contact with ruminants including cattle, while high cattle density counties had the highest disease rates [10]. Since *Campylobacter* spp. are abundant in cattle [11] and 1.1 million cattle were reportedly raised in Michigan in 2024 [12], there is potential for considerable spillover into the food chain and environmental reservoirs along with transmission to other animals within the agroecosystem [13, 14]. Continued monitoring of the molecular traits of strains circulating in different reservoirs and geographic locations is therefore critical for understanding *Campylobacter* epidemiology and estimating transmission risk to humans.

Genomic and molecular epidemiologic studies utilizing multilocus sequence typing (MLST) and whole-genome sequencing (WGS) have defined specific genetic lineages to be associated with cattle and other reservoirs. Strains classified as sequence type (ST)-982 and lineages that cluster within clonal complex (CC)-61 have been linked to cattle in several studies from different geographic locations [9,15,17]. In our prior Michigan studies, CC-61 was one of the most common lineages detected among cattle-derived *Campylobacter jejuni* from one beef and two dairy farms [18] and in patients with clinical infections [19]. ST-982-associated infections were also common in Michigan patients, particularly those who lived in rural areas, had been exposed to cattle and routinely consumed well water [19]. While the cattle-derived ST-982 strains were more commonly resistant to tetracycline, they had similar repetitive element sequence-based PCR (rep-PCR) fingerprint patterns as the human-derived ST-982 strains [18], raising questions about the pathogenic potential and transmissibility of this lineage.

Because of limitations with the seven-gene MLST scheme, discrepancies between specific lineages and host associations have been reported [15], prompting the development of more comprehensive genomic approaches. For instance, core genome MLST (cgMLST), which evaluates a set of 1,343 loci shared by ≥95% of strains, was developed to evaluate the population structure of *Campylobacter* spp. [20]. cgMLST data have enhanced understanding of C. *jejuni* evolution and were used to train a machine-learning algorithm to predict the source of clinical infections [21]. Computational mathematics, whether in machine-learning approaches or discrete clustering algorithms, has greatly advanced the field of genomic epidemiology by enabling the rapid clustering and analysis of large datasets [22,23]. Consequently, we applied pangenomic, machine-learning and hierarchical clustering approaches to compare a subset of 175 clinical *C. jejuni* genomes to 61 cattle-derived genomes recovered from Michigan during an overlapping 4-year period. Our findings highlight the importance of comparing strains that are circulating in both humans and reservoir species to detect emergent types and make predictions about transmission and virulence. When combined with epidemiological information, these data can be used to inform public health action.

## Methods

### Bacterial strains and DNA isolation

*C. jejuni* isolates were previously recovered via a prior epidemiologic study of zoonotic enteric pathogens in beef and dairy cattle [24]. Briefly, 220 faecal grab samples from dairy and beef operations in mid-Michigan were collected from July to August 2012 and cultured for *Campylobacter* [18]. Isolates were recovered by culture on tryptone soy agar containing 5% sheep blood, cefoperazone (20 µg), amphotericin B (4 µg) and vancomycin (20 µg per ml) overnight at 37 °C, in microaerophilic conditions as described [18,19, 25]. DNA was extracted using the Qiagen DNeasy Kit (Qiagen, Valencia, CA, USA), and each isolate was confirmed to be *C. jejuni* by PCR using a published assay [26]. For comparison, a subset of previously characterized human-derived *C. jejuni* strains was included; all strains were recovered from patients in Michigan presenting with campylobacteriosis between 2011 and 2014 [19]. Previously reported antibiotic susceptibility data were used for the cattle- and human-derived strains [18,25].

### WGS and sequencing analyses

The same methods that we previously used to sequence the genomes of the human-derived *C. jejuni* strains [27] and Shiga toxin-producing *Escherichia coli* [28] were applied to the cattle-derived *C. jejuni* strains. In brief, libraries were prepped with the Nextera XT library prep kit (Illumina, San Diego, CA, USA), and sequencing was performed on the MiSeq platform (Illumina) with 2×250 bp reads at the Michigan State University Research Technology Support Facility.

Trimmomatic v.0.36 [29] was used to trim adaptor sequences from raw reads, followed by *de novo* genome assembly using Spades v.3.15.2 [30]. FastQC v.4.10.1 [31], QUAST [30], and MultiQC v1.10.1 [32] were used to assess the quality of the reads and assembled genomes. All genomes were evaluated for quality via G+C content, completeness, N50 and number of contigs. Genomes with <400 contigs, N50 >10,000 bp, G+C content of 30.4–31.0 mol% and >85 mol% completeness were included, leaving 61 cattle- and 175 human-derived genomes for the pangenomic and phylogenetic analyses. Prokka v.1.14.6 [33] was used to annotate each genome with the ‘usegenus’ parameter available for *Campylobacter*.

Contigs were queried for the seven MLST loci with mlst v.2.19.0 [34], which uses the Bacterial Isolate Genome Sequence Database (BIGSdb) via PubMLST (https://pubmlst.org). Similarly, contigs were interrogated for a set of 1,343 cgMLST loci using cgMLST v.1.0 available through the PubMLST database [20]. Each assembly was screened for antimicrobial resistance genes and other determinants using the AMRFinderPlus v.3.10.23 pipeline [35], which is curated by the National Center for Biotechnology Information (NCBI). In addition, each strain was screened for the presence of *cmeB* variants, which encode efflux pumps that confer high levels of resistance to fluoroquinolones and macrolides, as described [36]. Those strains containing CmeB genes with <84.0% similarity to *cmeB* in reference strain NCTC 11168 (accession number AL111168.1) were classified as variants. Moreover, strains with an A103V point mutation in the L22 protein of the 50S ribosomal subunit were not classified as macrolide-resistant, as a prior study demonstrated that *Campylobacter* strains with the mutation are often susceptible [37]. As a result, only those strains possessing the A2075G mutation in the 23S gene were classified as having genotypic resistance to macrolides. Virulence genes were queried using ABRicate v.1.0.1 (https://github.com/tseemann/abricate) with the virulence factor database (VFDB) [38].

To screen for plasmid replicons, we used MOB-suite v.3.1.9, which can detect and reconstruct plasmids from short or long read assemblies using a reference-based approach with an existing database as described [39]. Briefly, MOB-recon uses a minimum sequence identity and coverage of 80% to reconstruct plasmids from assemblies, while MOB-cluster uses Mash to group plasmid sequences based on their nearest neighbour and assign MOB-cluster codes to each. Sequences under 1,000 bp are excluded. The MOB-typer tool predicts conjugation potential, size and host range. Based on MOB-recon clustering, we binned and tallied those plasmids with homology to *C. jejuni* plasmid sequences available in the NCBI. Representative plasmids were visualized and annotated with ProkSee [40] using BAKTA [41] with the following blast parameters: *e*-value (<1*^e^*^−10^), 100% alignment length cutoff and 80% identity cutoff. ProkSee was also used to predict plasmid G+C content, transfer, integration, excision stability, recombination and repair elements, while FastANI v.1.34 [42] was used to generate pairwise comparisons of different plasmids.

### Pangenome analysis and phylogenetic evolutionary reconstructions

Panaroo v.1.3 [43] was used to align the 61 cattle- and 175 human-derived *C. jejuni* genomes and all were evaluated for unique accessory characteristics. A multi-FASTA alignment of core genes was constructed with the MAFFT aligner in the ‘strict’ mode. The following default settings for clustering genes were used: 0.98 identity and length, family sequence identity of 0.7 paralogues and refining genes within a radius of 5,000 nt. The sequence alignment was used to generate a maximum-likelihood tree with the best-fit model automatically selected by ModelFinder [44] using IQTree v.2.2.0.3 [45] with 1,000 ultrafast bootstrap approximation. The generated best-fit consensus tree with 1,000 bootstrap support and rooted at the midpoint was visualized and annotated in the Interactive Tree Of Life (iTOL) v.6 [46]. Genomes that grouped together with 100% bootstrap support were considered part of a sequence cluster.

Unmerged raw reads from 19 sets of paired reads from the ST-982 strains from humans (*n*=12) and cattle (*n*=7) were used for a high-quality SNP (hqSNP) analysis. Lyve-SET [47], a pipeline that utilizes the VarSCAN SNP algorithm and the Lyve version of the SNP extraction tool (SET) to identify hqSNPs and omit low-quality SNPs, was used for this analysis with default parameters. As opposed to using cgMLST data, which identifies SNPs within a predefined set of core genes, Lyve-SET uses entire genomes to provide enhanced resolution. The programme also uses clustered SNP filtering to ensure that clusters of SNPs are not included, which are indicative of recombination events [47]. RAXML [48] was used to construct phylogenies based on the SNP distance matrix generated. The hqSNP tree was generated with 100 bootstrap replicates and annotated in iTOL v.6 [46] with metadata (e.g. source, year of collection, county and resistance phenotypes).

### Forecasting host source with a machine learning algorithm

To predict the source of the 175 human-derived *C. jejuni* strains, we input the cgMLST allelic profiles into the machine learning algorithm aiSource [21] that was trained on data in the BIGSdb [34] through PubMLST using cgMLST v.1.0 [20]. It uses the XGBoost algorithm, an example of supervised machine learning, and gradient boosting to generate decision trees. More specifically, the model was trained on the existing source data deposited in PubMLST for comparing cgMLST profiles to make predictions that could be tested on known datasets and refined. The source prediction was shown to have an ~84% forecast accuracy when trained using a global *C. jejuni* and *Campylobacter coli* dataset [21]; confidence in the forecast is reported in the output. Data were annotated in iTOL v.6 [46] on the maximum-likelihood tree generated from the core-gene alignment output from Panaroo v.1.3 [43] for visualization.

### Phylogeographic analysis of publicly available ST-982 genomes

Genome assemblies from the ST-982 strains were deposited in PubMLST. Using the query function in PubMLST in the BIGSdb [34], ST-982 strains with records containing complete ribosomal MLST (rMLST) data identified in the USA (*n*=1152) were added to our set of ST-982 strains from Michigan. The region (state), year, source and genomic metadata were downloaded from PubMLST when available. The complete set of records and paired genome data query was included for a global cgMLST analysis. Third-party applications available within PubMLST were used to evaluate the ST-982 population structure. Grapetree [49] generated a minimum spanning tree based on the cgMLST profiles, while ReporTree [50] created hierarchical clusters based on the Hamming distance of the cgMLST profiles generated with cg-dists [51]. The specific clustering pattern used for further analyses was selected by evaluating hierarchical clustering via silhouette analysis, which measures cohesion and separation of a clustering pattern to examine relationships within clusters [52]. The selection criteria included a clustering scheme with at least two informative clusters with the maximum average silhouette area and the minimum silhouette negative area within a clustering stability region defined in the output of ReporTree. Of the 1,152 ST-982 records in the database, 723 had complete data for state and year of isolation and were included in the downstream analyses. The phylogeny was visualized in GrapeTree, while the metadata and a Newick (.nwk) file can be further visualized with Auspice (https://auspice.us).

### Data analysis

Metadata were retrieved and managed using R [53], while vegan [54] and the brglm2, broom, car, gt and tidyverse (www.tidyverse.org/; accessed 1 March 2025) [55] R packages were used for the analysis and figure generation. The Welch’s t-test and Wilcoxon rank-sum test were used to evaluate differences in accessory-gene counts and genome size between groups. For the national analysis, regions were classified by the US Health and Human Services regional offices (https://www.hhs.gov/about/agencies/iea/regional-offices/index.html; accessed 25 May 2025). Univariate logistic regression was performed, and odds ratios (ORs) were calculated to estimate the magnitude of the association between region and allele cluster. Βeta diversity of allele profiles was assessed for alleles without missing data (*n*=423). Principal coordinate analysis (PCoA) of the cgMLST allele profiles using Jaccard distance was applied to evaluate allele profile diversity by cluster, while permutational multivariate ANOVA (PERMANOVA) evaluated the differences between groups.

## Results

### Lineage distributions vary among cattle- and human-derived *C. jejuni*

Among the 61 high-quality *C. jejuni* genomes from cattle, the *N*_50_ values ranged between 13.5 and 333.8 kb, while the genomes had between 21 and 376 contigs with an average of 93.4 contigs per genome (Table S1). The assembly size ranged from 1,613,666 to 1,914,118 bp, and the 61 genomes had an average G+C content of 30.5 mol%. Moreover, genome completeness ranged between 86.5 and 97.9%, averaging 91.5%. Similar genomic traits were observed among the 175 human-derived strains that were reported in our prior study [27].

**Fig. 1.**
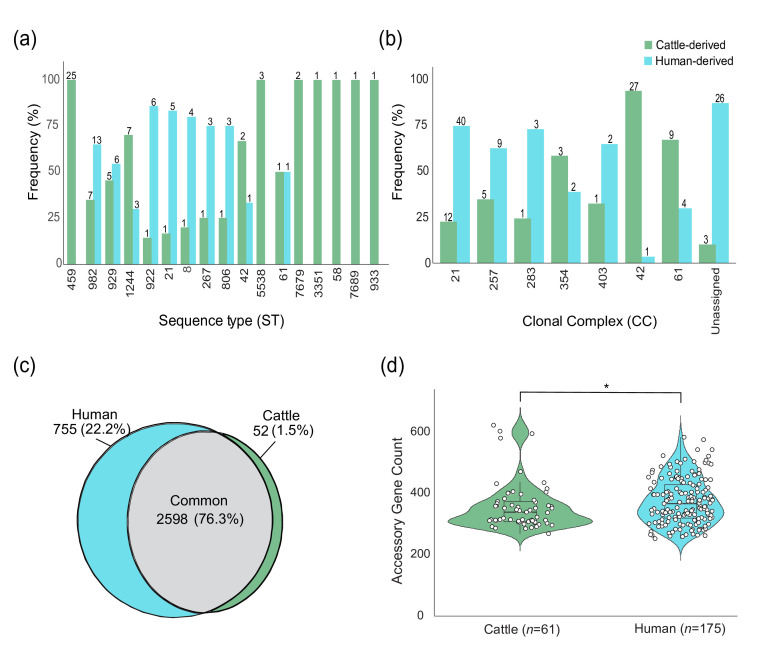
Lineage frequencies and pangenome characteristics in cattle- versus human-derived strains. Percent of cattle (green) and human (blue) strains representing each (a) ST and (b) CC. Actual counts are indicated above each bar; the percent was calculated using the total count for each ST or CC as the denominator. For simplicity, the STs and CCs that were only detected in the human-derived strains are not shown. (**c**) Venn diagram of genes identified only in the cattle-derived strains (green) as compared to those only found in the human-derived strains (blue). (**d**) Violin plot of the number of accessory genes among the cattle-derived strains (green) and human-derived strains (blue) with a box-and-whisker plot showing the mean (middle bar) and 95% confidence intervals (whiskers). *P=0.03, Wilcoxon rank-sum test.

Based on the 7 MLST locus sequences extracted per genome, the 61 cattle strains belong to 17 STs representing 7 distinct CCs that varied in frequency. Twenty-five of the 61 (41.0%) cattle-derived strains were classified as ST-459, which was not detected in the human-derived strains along with 6 additional STs (Fig. 1a). Among the ten STs that were found in both cattle and humans, a greater percentage of ST-1244 (11.5%; *n*=7) strains were recovered from cattle, while a lower percentage of cattle strains was classified as ST-982 (11.5%; *n*=7) and ST-929 (8.2%; *n*=5). Five additional STs were more common in human strains but were represented by only one cattle isolate each, whereas ST-61 was represented by a single cattle strain and one human strain. Altogether, the STs were assigned to seven different CCs with CC-42 and CC-61 predominating among the cattle strains (Fig. 1b). Among the 29 strains that could not be assigned to a previously defined CC via PubMLST, only three were from cattle. STs that were unique to humans [27] were omitted from the analysis.

### Fewer unique and accessory genes are present in the cattle genomes

Using Panaroo, we defined the pangenome of the 61 cattle strains for comparison to the pangenome of 175 human strains described previously [27]. Among the cattle strains, 2,519 distinct genes were detected from which 1,240 (49.2%) belong to the core genome and 201 to the soft-core genome (Table 1). The accessory genome was divided into shell genes (*n*=520) and cloud genes (*n*=558), which were detected in 15–95% and <15% of the genomes, respectively.

**Table 1.** Pangenome composition of cattle- and human-derived *C. jejuni* strains recovered from Michigan

	Combined pangenome (*n*=236)	Cattle strain pangenome (*n*=61)	Human strain pangenome (*n*=175)
**Genes**	**No. (%)**	No. (%)*	No. (%)*
**Core**	1,320 (38.8)	1,240 (49.2)	1,333 (39.9)
**Soft-core**	114 (3.3)	201 (8.0)	110 (3.3)
**Accessory**	1,971 (57.9)	1,078 (42.8)	1,895 (56.8)
Shell	499 (14.7)	520 (20.6)	489 (14.6)
Cloud	1,472 (43.2)	558 (22.2)	1,406 (42.1)
**Total**	3,405	2,519 (74.0)†	3,338 (98.0)†

*The percentage of each set of genes in cattle and human strains was calculated using the total number of genes from each source as the denominator.

†The total percentage of genes identified in the cattle and human strains was determined using the combined pangenome as the denominator.

Combining the 61 cattle-derived strains with the 175 human-derived strains contributed to a more diverse pangenome with 1.4 times more total genes (*n*=3,405) in all 236 strains relative to the cattle pangenome alone. The cattle pangenome only represented 74.0% of the total pangenome, whereas the human pangenome represented most (98.0%; *n*=3,338) of the 3,405 genes. Among these genes, only 52 were unique to the cattle strains (Table S2) compared to 755 that were unique to the human strains (Table S3). The remaining 2,598 genes were shared between groups (Fig. 1c). Although no difference in the average number of accessory genes was observed between the cattle (*n*=357) and human (*n*=374) strains (Welch’s t-test, *P*=0.12), variation in the distribution of accessory genes was detected between groups (Wilcoxon rank-sum test, *P*=0.03; Fig. 1d). Intriguingly, 61.5% (*n*=32) of the 52 unique genes found in the cattle strains were only in one ST-58 strain, TW17736, and most encoded hypothetical proteins (Table S2). The 20 remaining unique genes were found in 24 other cattle strains.

### Tetracycline and *β*-lactam resistance determinants are common in cattle strains

**Fig. 2.**
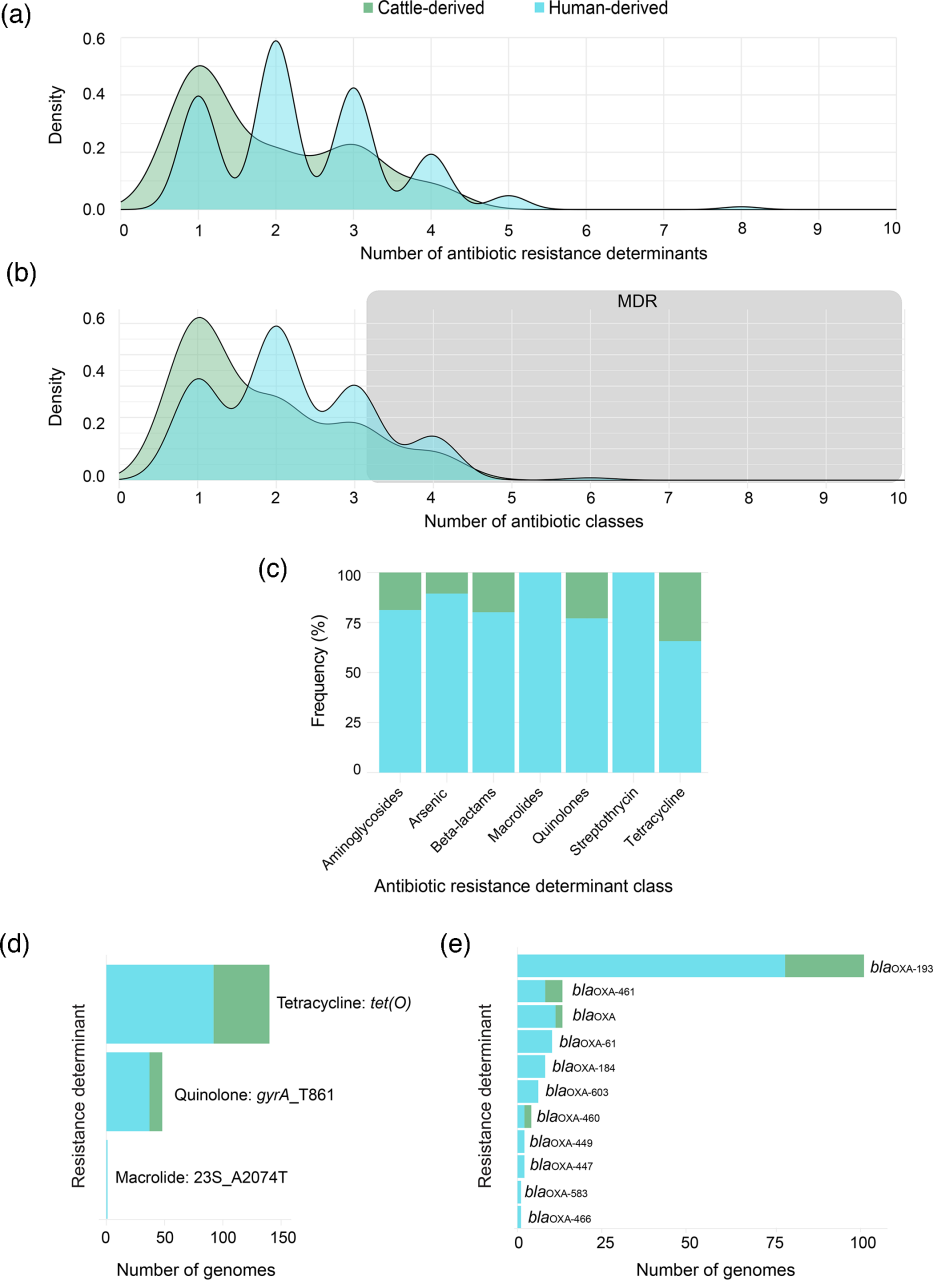
Antimicrobial resistance determinants stratified by strain source. Density plot showing the (a) number of resistance determinants and (b) antibiotic classes in the cattle (green) and human (blue) genomes. The grey background in panel (b) represents genomes with MDR. (**c**) The percentage of antibiotic classes linked to resistance determinants that were detected within the cattle (green) and human (blue) genomes. (**d**) The number of genomes with specific determinants that confer resistance to tetracyclines, quinolones and macrolides (e) as well as the *β*-lactams stratified by source.

Most (*n*=59; 96.7%) of the cattle strains possess at least 1 antibiotic resistance determinant, whereas the human strains have an average of 3, with 131 strains having 2 or more (Fig. 2a). Multidrug resistance (MDR) was detected in both strain populations; however, a greater proportion of human strains (40.0%; *n*=70) had genes conferring resistance to three or more antibiotic classes relative to the cattle strains (29.5%; *n*=18) (Fig. 2b). Although there were fewer cattle strains, which represented only 26% of the 236 strains, ≥20% of the *β*-lactam, quinolone and tetracycline resistance determinants were identified in these strains (Fig. 2c). The MDR strains from cattle predominantly carried genes conferring resistance to quinolones, tetracyclines and *β*-lactams, whereas most human MDR strains had genes encoding resistance to heavy metals (*arsP* or *acr3*), tetracycline and quinolones.

In all, 34.3% (*n*=48) of the 140 strains with tetracycline resistance genes were cattle-derived, with 26 (42.1%) of these strains possessing *tet(O)* that encodes a ribosomal protection protein (Fig. 2d). A quinolone resistance mutation, which modifies gyrase (*gyrA*_T86I), was identified in both the cattle and human strains, while a single human genome contained a mutation in the 23S ribosomal subunit conferring resistance to macrolides. None of the recently described *cmeB* variants encoding high levels of fluoroquinolone and macrolide resistance [36], however, were detected in any of the strains. All harbour a CmeB gene with 94.6–100% similarity to *cmeB* from reference strain NCTC 11168. Multiple genes encoding *β*-lactam resistance were also detected. While 161 (68.2%) genomes had genes encoding *β*-lactamase production, only 19.8% (*n*=32) were detected in the cattle strains (Fig. 2e). Indeed, the diversity of *β*-lactamase genes was greater in the human strains with 11 different alleles, as compared to only 4 alleles in the cattle strains. Among the determinants that were found in strains from both sources, *bla*_OXA-193_ predominated followed by *bla*_OXA-461_, *bla*_OXA_ and *bla*_OXA-460_*.*

Importantly, there was not always concordance between the genotypic and phenotypic resistance profiles. For example, a single ST-1244 MDR cattle-derived strain (TW17747) with phenotypic resistance to macrolide, lincosamide and ketolide (MLK) antibiotics as well as fluoroquinolones and tetracyclines (Table S1) [18] lacked genes for MLK resistance. Similar inconsistencies were observed for one of the human-derived strains, TW19135, which had the same MDR profile and lacked known MLK resistance determinants as noted [27]. The remaining 60 cattle strains, however, had genomic predictions that were concordant with the phenotypic susceptibilities.

### Type IV secretion system genes are more common in cattle genomes

**Fig. 3.**
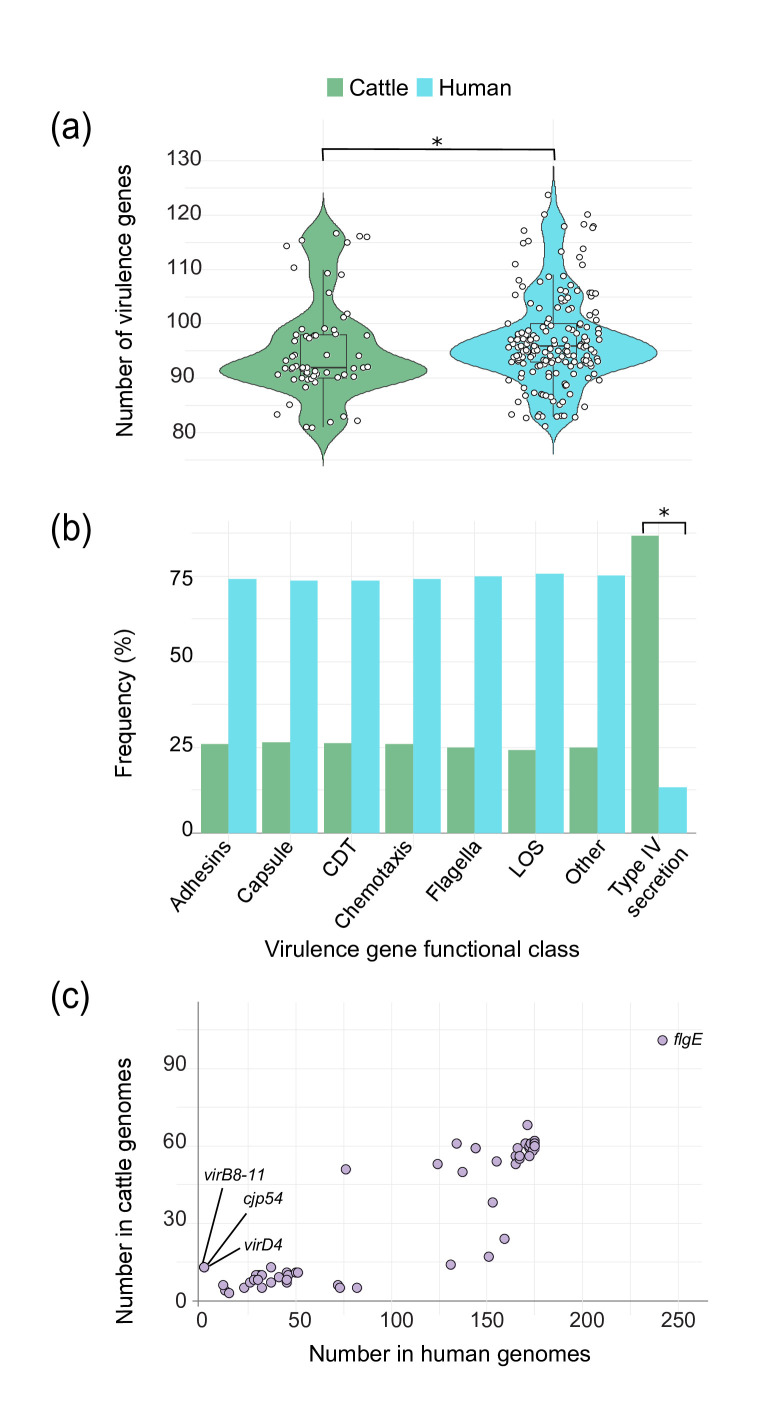
Number and type of virulence genes detected in cattle versus human genomes. (**a**) Violin plot showing the number of virulence genes among the cattle (green) and human (blue) genomes. The box-and-whisker plot displays the 95% confidence interval and mean (middle black bar) by source (*P=0.04, Wilcoxon-rank sum test). (**b**) The percentage of virulence gene classes detected in the cattle versus human genomes. *P<0.001, chi-square test. CDT, cytolethal distending toxin; LOS, lipooligosaccharide. (**c**) Number of virulence genes identified in the cattle genomes (x-axis) and the human genomes (y-axis); genes associated with type IV secretion systems (*vir8-11*, *virD4* and *cjp54*) are labelled as well as *flgE* (flagella), which was found in multiple copies in some genomes.

Although the cattle and human strains have a similar number of virulence genes per genome (Welch’s t-test, *P*=0.25), the distribution of these genes differed in the two strain populations (Wilcoxon rank-sum test, *P*=0.04; Fig. 3a). Stratifying by the functional class of each virulence gene also uncovered differences in the proportion of genes between the two groups. Notably, the cattle strains have a greater proportion of type IV secretion system genes than the human strains (chi-square test, *P*≤0.001; Fig. 3b). Plotting the number of virulence genes in the cattle versus human genomes shows the presence of specific type IV secretion system genes, *virB8-11*, *virD4* and *cjp4*, to be more prevalent in cattle strains (Fig. 3c). Although all genomes possess *flgE*, which is an essential gene that codes for the unipolar flagellar hook protein, more than one copy of *flgE* was detected in 107 genomes from both sources (cattle *n*=40; human *n*=67). Using the VFDB [38], these 107 genomes possess genes that are homologous to FlgE flagellar hook proteins, NP_281265 and NP_282855 [56], with >83% identity.

### Core-gene phylogenetic reconstruction identifies highly related strains circulating in cattle and humans during the same time period

**Fig. 4.**
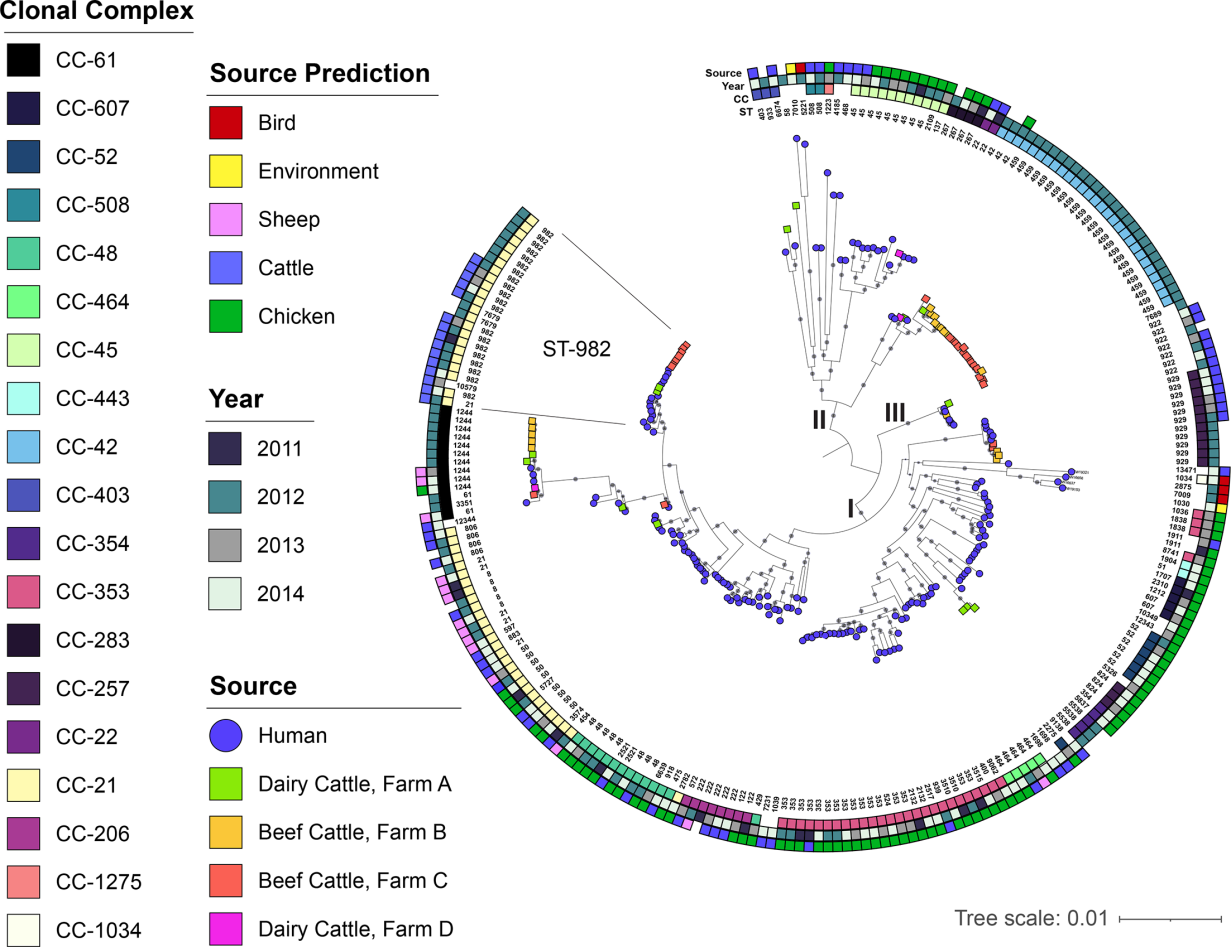
Core gene maximum-likelihood phylogeny showing the degree of relatedness among the human and cattle strains. The maximum-likelihood phylogeny was constructed using 1,320 core genes and was rooted at the midpoint between the 2 longest branches; nodes with bootstrap support values of 100% are shown as dark grey circles. Three sequence clusters, designated as I, II and III, were identified. Coloured shapes at the branch tips indicate which strains are from humans (blue circle) or different cattle herds (coloured squares). The numbers at the end of each branch represent the accession numbers, while the multilocus STs are listed in the innermost circle. Moving towards the outside of the circle, the innermost coloured squares represent the CC followed by the year of isolation and the predicted source of infection for the human-derived strains. The cluster of genomes belonging to the ST-982 CC is labelled.

A midpoint-rooted maximum-likelihood phylogeny based on the alignment of 1,320 core genes revealed 3 sequence clusters that grouped together with 100% bootstrap support (Fig. 4). One cluster (I) comprised the majority (*n*=172) of the strains examined and contained multiple subclusters of related strains. The other two clusters (II and III) contained 56 and 8 strains, respectively. The 61 cattle strains were distributed throughout the phylogeny and were found in each of the three clusters. Many of the cattle strains were grouped together despite being recovered from four different herds. The same was true for human strains recovered from different patients. The strains were sorted by CCs within the phylogeny, though some STs (e.g. STs 21, 353 and 429) of a CC were found on different branches. The ST-58 cattle strain (TW17736) with the most (61.5%) unique genes was a singleton within the second cluster.

Plotting the date of collection (year) onto the phylogeny showed that strains from the same years did not always cluster together (Fig. 4). Since most cattle strains were recovered in only one of the four years, several of those from 2012 grouped together. Nonetheless, these clusters often came from cattle in different herds. For example, strains belonging to ST-459 (CC-42) from 2012 clustered together even though they were recovered from cattle at two different beef herds and one dairy herd. The same was true for the ST-982 cluster of CC-21 strains as well as the ST-1244 (CC-61) and ST-929 (CC-257) clusters, each of which included strains from different herds. Several human strains from later years (2013–2014) also grouped with cattle strains in each cluster, highlighting the persistence of some lineages in this geographic location.

### Source predictions implicate chicken and cattle in most human infections

To predict the source of the 175 human-derived strains, we ran the cgMLST profiles through aiSource [21]. This analysis identified the potential source of most human infections to be cattle (*n*=66) and chickens (*n*=88); however, links to the environment (*n*=2), sheep (*n*=14) and birds (*n*=4) were also found (Table S4). The source for one strain could not be predicted.

Mapping the predicted sources onto the core gene phylogeny (Fig. 4) uncovered relationships with specific clusters and confirmed several prior associations with certain CCs. Because of the enhanced ability of the aiSource programme to predict *C. jejuni* source attribution relative to the conventional seven-gene MLST scheme [21], not all strains of the same ST were predicted to have the same source. For instance, the highly related ST-353 (CC-353) strains recovered in multiple years were attributed to chicken, except for one strain that was predicted to be linked to cattle. The same was true for ST-50 (CC-21), which comprises highly related strains from multiple years that were predicted to have different sources including chicken, cattle and sheep.

Among the three core gene sequence clusters that contained both cattle and human strains, the predicted source of the human strains was not always cattle. For example, the cluster of two ST-42 (CC-42) cattle-derived strains from one dairy and one beef herd was related to one human-derived isolate that was predicted to originate from chickens. The same was true for the cluster of four ST-267 strains, which contained one strain from a dairy cow and three human-derived strains that were predicted to originate from chickens. Similarly, the highly related cluster of ST-1244 (CC-61) strains comprised strains from four cattle herds that grouped with four human strains predicted to originate from sheep (*n*=3) and chickens (*n*=1). Differences in strain clustering by farm were also observed as three strains from a dairy farm belonging to ST-5538, which was not identified in beef farms, were most closely related to a group of human-derived strains with chicken source predictions.

Clustering of cattle- and human-derived strains that were predicted to come from cattle was also observed (Fig. 4). The cluster of CC-21 strains containing ST-982 (*n*=20), ST-7679 (*n*=2), ST-21 (*n*=1) and ST-10579 (*n*=1), for instance, was mixed with cattle-derived strains from one dairy (*n*=2) and one beef herd (*n*=7) as well as human strains (*n*=15). Within this cluster, the human strains were all predicted to have originated from cattle across a span of 4 years. The same was true for the ST-929 (CC-257) cluster of 11 strains, which contained cattle strains from 2 beef herds (*n*=5) that were highly related to 6 human strains recovered over a 3-year period with cattle source predictions.

### Human- and cattle-derived ST-982 strains are distinct

**Fig. 5.**
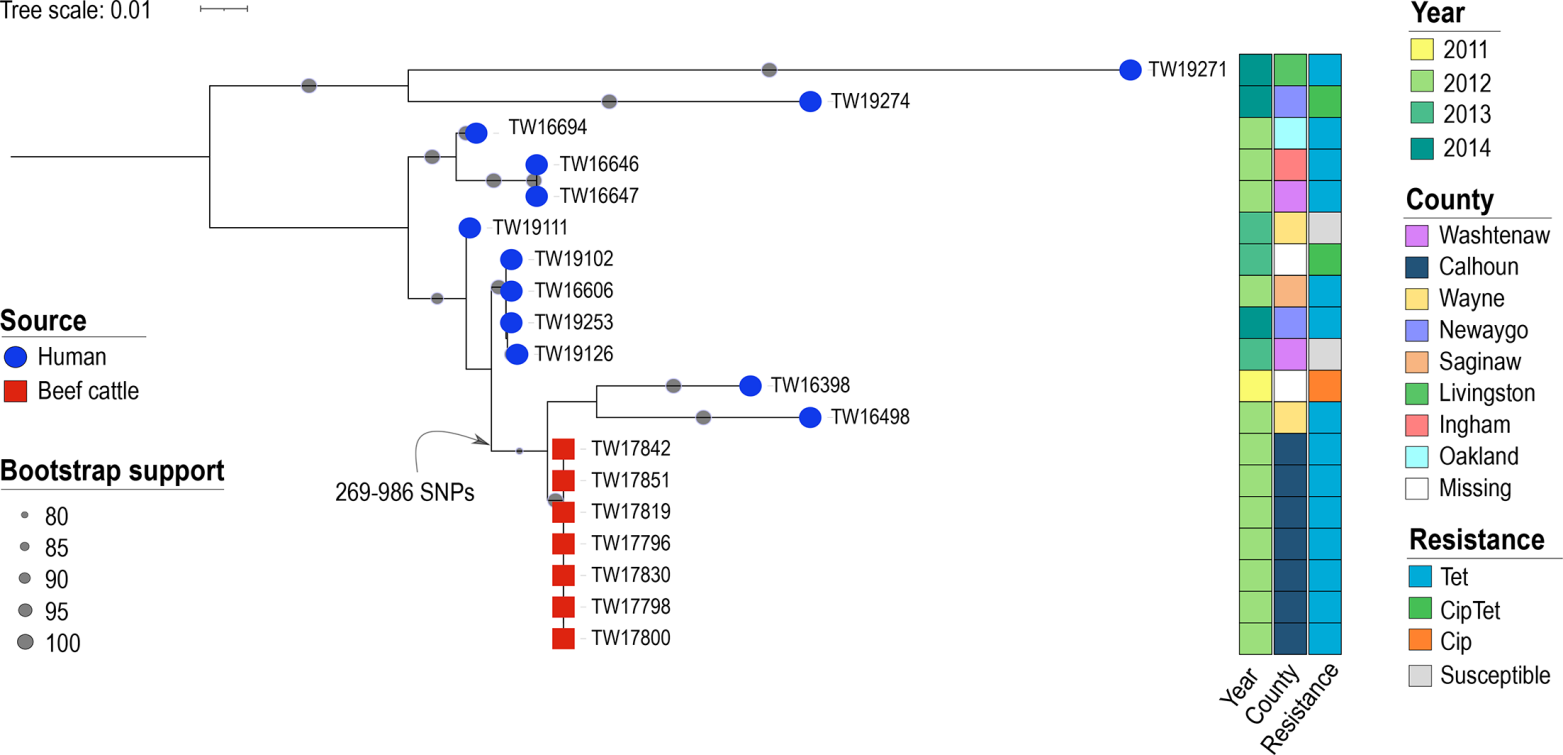
Relationships among human- and cattle-derived ST-982 strains based on an hqSNP analysis. The maximum-likelihood phylogeny was constructed based on hqSNP distance rooted at the midpoint between the two longest branches. Grey circles represent bootstrap support values for each branch. Branch tips are labelled with coloured shapes to indicate the strain source: human-derived (blue circle), beef-cattle derived (red square). At the end of each branch is the accession number with the coloured squares representing the year of isolation, resistance phenotype and county. Tet, tetracycline; CipTet, ciprofloxacin and tetracycline; Cip, ciprofloxacin.

Given the clustering of cattle and human ST-982 strains within the core-gene phylogeny and the prediction that cattle was the source of ST-982 human infections, we compared hqSNPs in the shared loci using Lyve-SET [47] to evaluate relatedness. Nineteen ST-982 strains, including 7 from beef cattle and 12 from humans, were selected based on their placement in the phylogeny (Fig. 4). Notably, the hqSNP analysis identified several clusters of highly similar human strains and only one cluster of cattle strains (Fig. 5). Several singletons were also observed among the human strains and are located on distinct branches of the hqSNP phylogeny. All seven cattle strains had identical hqSNP profiles (0 SNP differences within their shared loci) and grouped within a cluster that was most closely related to two human strains, TW16398 and TW16498. Of note, the accessory genes varied in this subset of strains, but the plasmid profiles were the same. The node separating these two groups, however, indicates that they differ by >260 SNPs. It is noteworthy that human strain TW16498 was recovered in the same year as the cattle strains and expressed resistance to tetracycline as well. The human case, however, was diagnosed with campylobacteriosis in a different county than the feedlot location.

### Plasmid distribution varies by genome and ST-982 strains harbour unique plasmid combinations

Because genes encoding type IV secretion systems were more abundant in cattle genomes (Fig. 3b) and are associated with *C. jejuni* plasmids [57], we screened for and extracted plasmid replicons from the assembled genomes. In all, 440 plasmid replicons were detected that belonged to 12 different MOB-clusters (Table S5). All but one strain has at least one plasmid. An average of two plasmids per genome was observed, though some have three (*n*=36) or four (*n*=12). No difference in the number of plasmids was observed between the cattle and human genomes (Welch’s t-test, *P*=0.12).

As described previously [58], the plasmids were classified into one of the following four plasmid types: type 1 (tetracycline resistance plasmid, pTet), type 2 (*C. coli* plasmids), type 3 (pVir plasmids) and type 4 (short plasmids<6000 bp). All plasmids >100 kb were classified as megaplasmids. Among the 236 strains, a plasmid belonging to one MOB-cluster, AG887, predominated and was found in 98.3% of genomes (Table 2). This plasmid ranges in size from 35.7 to 151.9 kb and exceeds 100 kb in 167 strains. It was predicted to be non-mobilizable in all genomes with homology to CP023447, a 103 kb megaplasmid originally described in a *C. jejuni* strain from poultry meat sampled in Brazil [59].

**Table 2.** Number and type of plasmids detected in *C. jejuni* genomes from humans and cattle

MOB-cluster ID	No.	Size range (bp)	Mobility prediction (n)	Accession of nearest neighbour(s)	Nearest neighbour host	Plasmid type*	Classification
AB469	31	24,323 to 38,811	C	CP017231	*C. jejuni*	pFORC46.2	pVir
AB625	3	5,081 to 7,454	NM	MK251542	*Glaesserella parasuis*	Type 4	Type 4
AB915	2	3,390 to 3,409	NM	CP027488	*Staphylococcus aureus*	Type 4	Type 4
AC320	75	8,432 to 48,904	C (54), M (18), NM (3)	CP013035, AY394560, AY394561, AY714214, CP001961, CP002030, CP007180, CP007750, CP007752, CP010073, CP017416, CP017861, CP017869, CP022471, CP043764, KJ646012, NC_022354	*C. jejuni, C. coli*	Type 1	pTet
AC321	18	22,584 to 79,062	C (9), NM (9)	CP013117, CP013033, CP017854, CP017877, CP022078	*C. jejuni, C. coli*	Type 1	pTet
AC439	8	4,494	M	CP045047, MH634989	*C. jejuni, C. coli*	Type 4	Type 4
AC498	1	3,724	NM	AB211496	*Campylobacter lari*	Type 4	Type 4
AC501	1	2,046	NM	NC_008438	*C. jejuni*	Type 4	Type 4
AD894	1	3,999	NM	NC_021493	*C. jejuni*	Type 4	Type 4
AE015	14	10,567 to 38,714	C (8), NM (6)	CP038864, CP014746, NC_008770, CP010074	*C. jejuni*	Type 3	pVir
AE190	54	25,049 to 142,383	C (3), NM (51)	CP014345, CP014743, CP014745. CP017417, CP018901, CP025282, CP044174	*C. jejuni, C. coli*	Type 3	pVir
AG887	232	35,730 to 151,984	NM	CP023447	*C. jejuni*		Unknown

Mobility prediction abbreviations: C, conjugative; M, mobilizable; NM, non-mobilizable.

*Classification (type) system adopted from Marasini *et al.* [58] for types 1–4; type 4 plasmids were previously classified based on having fewer than 6,000 bp.

The next most common plasmids were classified as pTet (39.4%) belonging to MOB-clusters AC320 and AC321, which were found in 75 and 18 genomes, respectively. pVir type 3 plasmids representing MOB-clusters AE015 (*n*=14) and AE190 (*n*=54) were also common. Three of the AE190 pVir-like plasmids were megaplasmids exceeding 100 kb, and each was found in a different human-derived strain. While six distinct type 4 plasmids were found within 16 genomes, no type 2 plasmids were detected.

Of note, plasmids belonging to MOB-cluster AB469, which represent conjugative plasmids between 24.3 and 38.8 kb, were detected in 31 genomes. This latter group of plasmids has homology to a different pVir-like plasmid (CP017231, pFORC46.2 [60]), originally isolated from a Korean patient with campylobacteriosis (NCBI BioSample SAMN05411282). One cattle strain possessed this AB469 pVir-like plasmid as well as the two other pVir plasmids representing MOB-clusters AE015 and AE190. Similarly, 12 strains had AB469 along with 1 of these 2 pVir plasmids; all but three of these strains were from cattle. Seven of these 12 strains also possessed pTet (AC320).

**Fig. 6.**
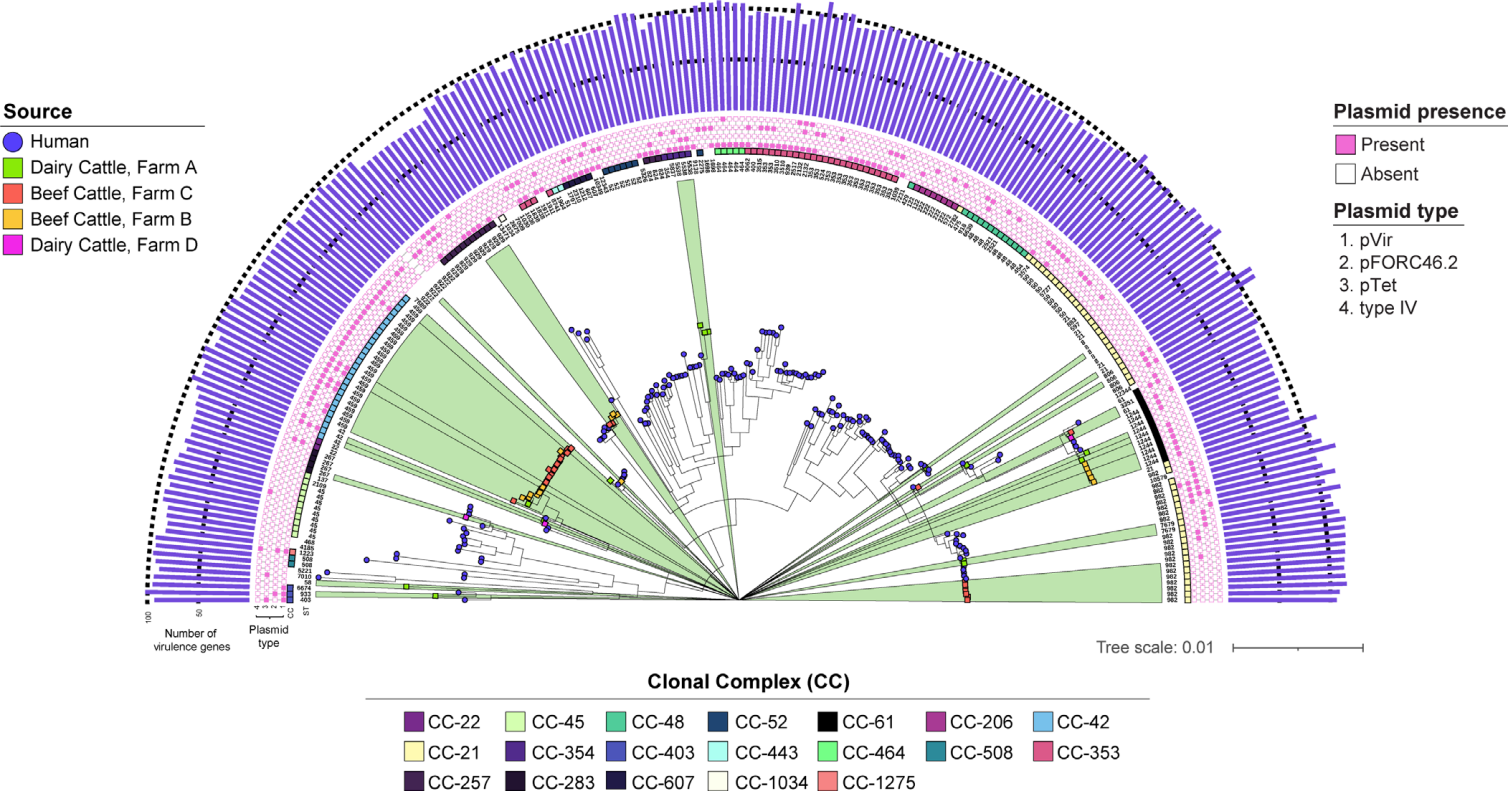
Maximum-likelihood core gene phylogeny showing the distribution of virulence genes and plasmids among human and cattle genomes. A condensed version of the same maximum-likelihood phylogeny (Fig. 4) based on 1,230 core genes is shown. The coloured shapes at the branch tips indicate which strains are from humans (blue circle) or cattle at four different herds (coloured squares). The numbers at the end of each branch (innermost circle) represent the multilocus STs followed by coloured squares representing the different CCs. The inner four rings show the presence (pink square) and absence (open square) of four common plasmids labelled 1–4 corresponding to pVir, pFORC46.2, pTet and type IV, respectively.

**Fig. 7.**
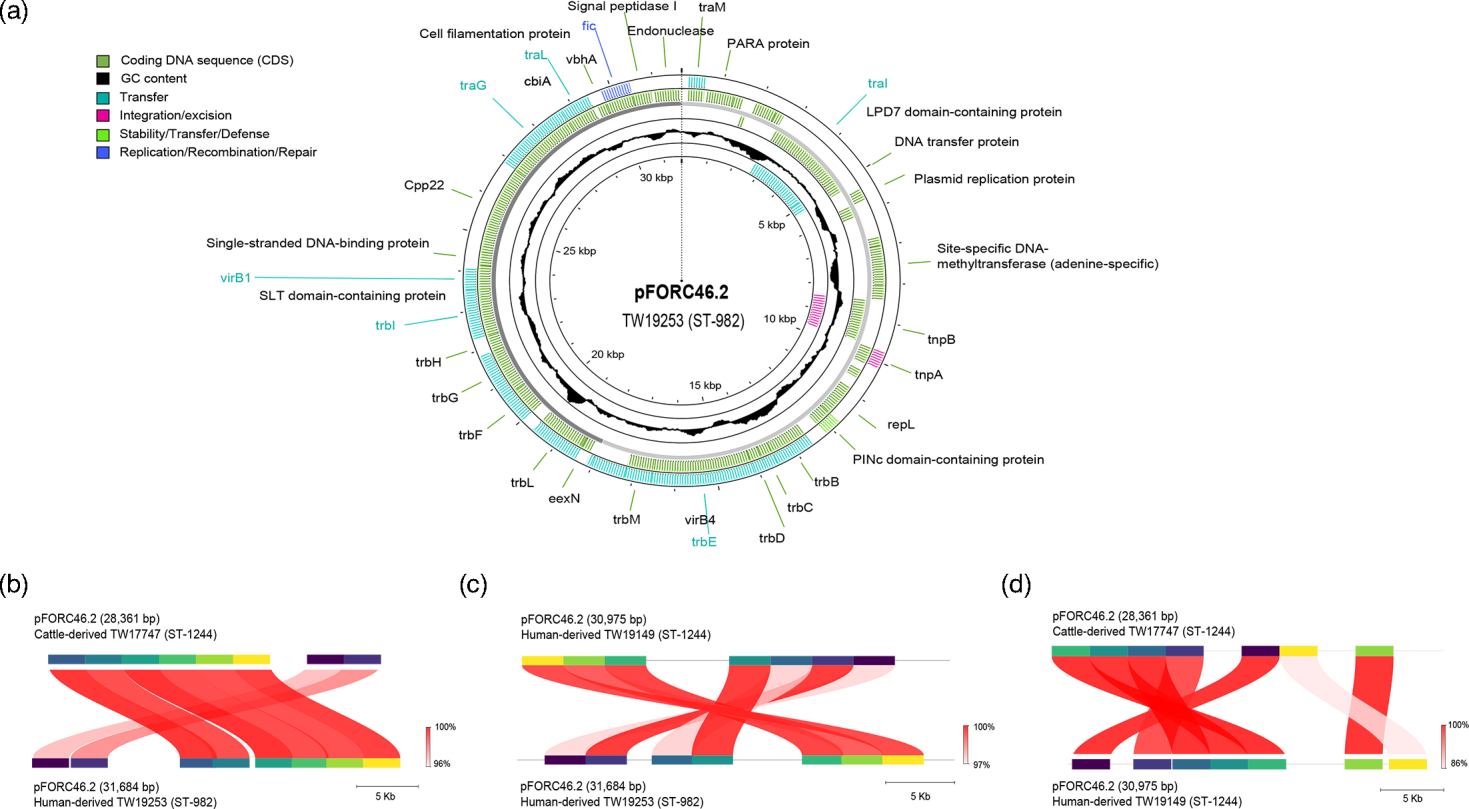
Comparative sequence analysis of pFORC46.2 (AB469) plasmids from *C. jejuni* strains recovered from cattle and humans. (**a**) Map of the pFORC46.2 plasmid from a ST-982 human-derived strain (TW19253). The gene and coding DNA sequence (CDS) categories representing different categories are colour-coded by function, while the G+C content is represented by the black line. Pairwise comparisons show sequence similarity between the TW19253 pFORC46.2 plasmid (bottom) and two CC-61 (ST-1244) strains from a (b) cow (TW17747) and (c) patient with campylobacteriosis (TW19149). (d) A comparison between the two CC-61 plasmids. The scale shows the per cent similarity gradient for each set of plasmid comparisons.

Mapping the type of plasmid identified within each genome onto the midpoint-rooted core gene maximum-likelihood phylogeny uncovered differences in the distribution of some plasmids (Fig. 6). For instance, pTet predominates among the cattle-derived CC-42 (ST-459) strains but is also found on different branches of the phylogeny within CCs 353, 61 and 21. Each of these CCs comprises strains with pTet as well as one of the three pVir-like plasmids. Among the nine cattle-derived strains containing a pVir (AE015, AE190) plasmid plus the pVir-like pFORC46.2 (AB469) plasmid, six belong to CC-42, two belong to CC-354 and one belongs to CC-403. The distribution of the pFORC46.2 plasmid is notable, as it was most common in the ST-982 (CC-21) and CC-61 strains. Seven ST-982 human-derived genomes have pFORC46.2, with one harbouring another pVir and pTet plasmid, as do several CC-61 strains from both humans and cattle. To determine how similar these pFORC46.2 plasmids are among strains from both sources, a plasmid map of the 31,685 bp AB469 plasmid was constructed in a representative human-derived ST-982 (TW19253) strain (Fig. 7a). This plasmid possesses genes for conjugative transfer, stability and replication as well as type IV secretion, which were described previously [58]. It has >95% sequence similarity to the pFORC46.2 plasmids from two CC-61 strains recovered from a cow (Fig. 7b) and a patient with campylobacteriosis (Fig. 7c). The latter two plasmids are also highly related to each other (Fig. 7d), suggesting that similar strains and plasmid types are co-circulating in cattle and humans in this geographic location.

### ST-982 sub-populations demonstrate geographic separation and diversification

**Fig. 8.**
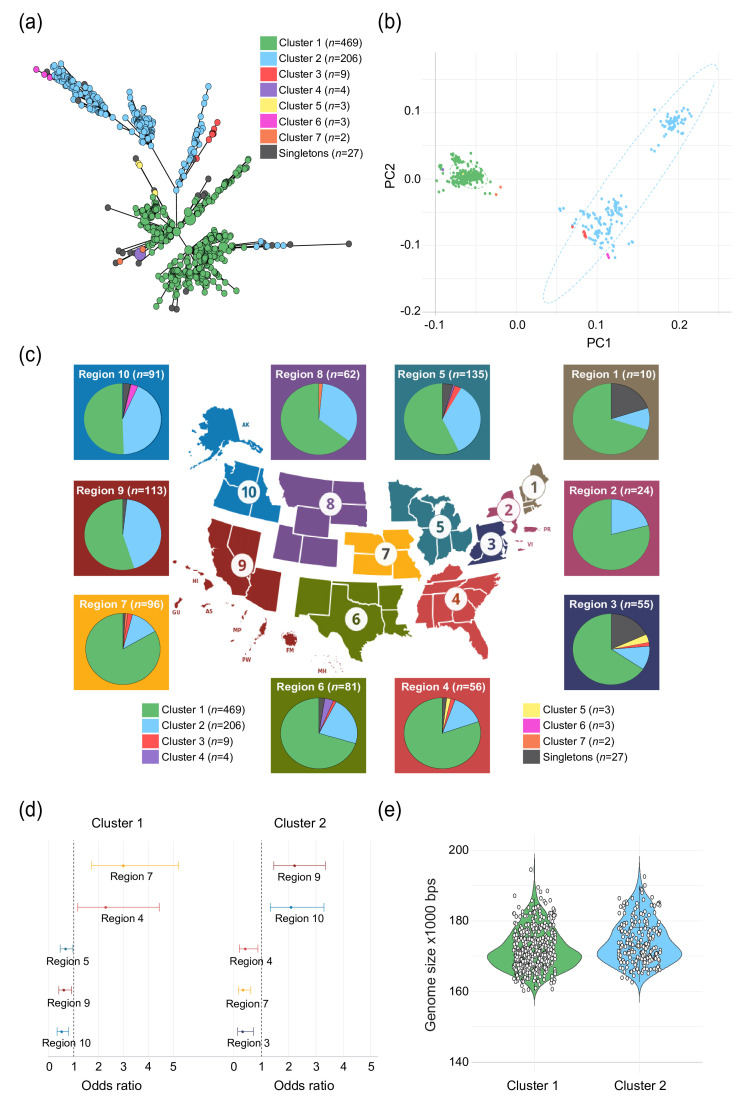
Characterization of publicly available ST-982 genomes representing *C. jejuni* strains recovered in the USA. (**a**) Minimum-spanning tree (MST) based on cgMLST showing seven clusters. (**b**) PCoA based on cgMLST profiles using Jaccard distances. (**c**) Map of the different US regions with pie charts that show the distribution of clusters identified in the MST analysis by region. The background colours of each pie chart pertain to those shown in each region. (**d**) Forest plots of ORs that highlight the magnitude of the associations between regions and cluster distributions. ORs were calculated using univariate logistic regression, though only those regions with significantly different distributions are shown. (**e**) Violin plot showing the genome size for each strain by cluster.

Since the ST-982 strains from cattle and humans in Michigan are highly related with similar genomic features, we compared them to a larger set of publicly available ST-982 genomes (*n*=723) from multiple sources and locations in the USA. Using cgMLST and hierarchical clustering based on core-gene Hamming distances, 7 clusters of ST-982 genomes were resolved along with 27 singletons. The minimum spanning tree generated from the cgMLST profiles demonstrated an unbalanced topography with a central star-like shape and additional bifurcations arising from nodes further from the centroid (Fig. 8a). The seven clusters were arbitrarily named clusters 1–7. Among the 14 most closely related ST-982 genomes from cattle and humans in Michigan examined by hqSNP analysis herein (Fig. 5), all belong to cluster 1. Forty-one additional ST-982 publicly available genomes were from Michigan and had been recovered from either cattle (*n*=39) or chicken meat (*n*=2) between 2015 and 2023 (Table S6). These genomes mostly belong to cluster 2 (*n*=22) and cluster 1 (*n*=15), though one strain is part of cluster 3 and three are singletons.

Among all 725 ST-982 genomes, most (*n*=469; 64.9%) grouped within cluster 1 or cluster 2 (*n*=206; 28.5%). To confirm the clustering defined in the minimum spanning tree, a PcoA of Jaccard distance based on the presence and absence of complete alleles (*n*=423) was used. Distinct separations were observed for cluster 1 and cluster 2 (PERMANOVA, *P*=0.001; Fig. 8b). The smaller clusters grouped together; however, they were not distinguishable from clusters 1 or 2 when all ST-982 genomes were included in the analysis.

To evaluate the phylogeography of ST-982, we stratified the distribution of clusters by the US regions (Fig. 8c). Differences in the number of ST-982 strains were observed by region, with some clusters predominating in specific regions relative to others. Although cluster 1 predominated in all nine regions, they were significantly more common in region 7 (*n*=80; 83.3%) relative to all other regions [OR: 2.3, 95% confidence interval (CI): 1.71–5.21; Fig. 8d)]. The same was true for region 4 (OR: 2.3; 95% CI: 1.16–4.44), which comprised 19 (80.4%) cluster 1 ST-982 strains. By contrast, cluster 1 strains were less likely to be recovered from regions 5 (OR: 0.7; 95% CI: 0.45–0.97), 9 (OR: 0.6; 95% CI: 0.40–0.91) and 10 (OR: 0.5; 95% CI: 0.32–0.79) as compared to all other regions. Cluster 2 was more prevalent in regions 9 (OR: 2.2; 95% CI: 1.46–3.34) and 10 (OR: 2.1; 95% CI: 1.33–3.28) but was less common in regions 3 (OR: 0.3; 95% CI: 0.13–0.71), 4 (OR: 0.4; 95% CI: 0.20–0.88) and 7 (OR: 0.3; 95% CI: 0.18–0.61).

Although cluster 6 was limited to three strains, all three were unique to region 10. Moreover, some smaller clusters (e.g. 4, 5 and 7) were detected in regions comprising states that are adjacent to those in different regions. For example, the three cluster 5 strains were recovered from Pennsylvania and Maryland (region 3) as well as North Carolina (region 4), which are in the same geographic vicinity despite the region classification. It is notable that genomes belonging to clusters 1 (1.72 Mbps, average genome size) and 2 (1.74 Mbps, average genome size) could be further differentiated based on their average genome size, with cluster 2 strains having larger genomes, on average, than cluster 1 strains (Welch’s two-sample t-test t, *P*=≤ 0.001) (Fig. 8e).

## Discussion

The collective use of pangenomic, machine learning and hierarchical clustering approaches has allowed us to more comprehensively compare *C. jejuni* genomes from Michigan patients with campylobacteriosis to those from dairy and beef cattle collected during an overlapping time period. Unique ST/CC distributions were observed in strains from both sources; however, the cattle strains were less diverse with fewer unique genes and accessory genes that are important for adaptation [61]. The cattle strains also had distinct virulence gene and plasmid combinations. Although we only screened for *C. jejuni* in cows at four herds, which may not be representative of the entire cattle population in Michigan, the findings are consistent with prior studies linking horizontal gene transfer and gene loss to cattle adaptation [13,16].

A diverse array of antimicrobial resistance genes, virulence genes and plasmids was detected in both the cattle and human genomes. Significantly more virulence genes and a higher frequency of MDR were observed in the human versus cattle strains; however, some differences were observed by source. While multiple *β*-lactamase (*bla*) genes were detected in genomes from both sources, with *bla*_OXA-193_ predominating overall, the human strains had a greater number of *bla* alleles than the cattle strains. This difference is likely due to the enhanced pangenomic diversity of the human strains examined, as many patients had reported a recent history of travel that was independently associated with increased diversity [27]. Phenotypic resistance to *β*-lactam antibiotics is common in *C. jejuni* and has been linked to chromosomal mutations in the promoter region of class D *β*-lactamase genes (e.g. *bla*_OXA-61_) [62]. As a result, this class of drugs is not typically used to treat campylobacteriosis. Nonetheless, widespread use of *β*-lactam antibiotics in livestock selects for these pathogens in agricultural environments and reservoir species [63], thereby promoting adaptation and persistence while increasing the likelihood of food product contamination.

Forty-eight (78.7%) of the 61 cattle strains also possess the tetracycline resistance gene, *tet(O)*, which is often found on conjugative pTet plasmids that have been linked to the dissemination of antibiotic resistance in *C. jejuni* [64]. This finding is less surprising since we have previously observed high rates of tetracycline resistance in both human [25] and cattle [18] strains as well as an association between resistance and rural residence, history of cattle contact and infection by cattle-associated *C. jejuni* lineages (i.e. ST-982) [19,25]. Of the 48 cattle strains with *tet(O)*, 11 (22.9%) were classified as MDR both genotypically and phenotypically [18,27]. Even though only 2 of these 11 (18.2%) MDR cattle-derived strains possess pTet, the ease of plasmid transfer, potential for carrying other resistance genes and its widespread distribution highlight the need for continuously monitoring resistance determinants in *Campylobacter* spp. Indeed, entry of these plasmids into the human gut microbiome is a serious concern, as they can be acquired by other species during infection [65], contributing to the emergence and widespread dissemination of resistance.

The increased abundance of genes encoding type IV secretion system proteins (*virB8-11*, *cjp54* and *virD4*) in the cattle versus human strains is noteworthy and consistent with reports of high gene frequencies in dairy cattle isolates from Korea [66]. This finding suggests that the cattle strains may have a greater propensity for conjugation, which could enhance fitness, adaptation and dissemination of plasmid-associated virulence and resistance genes. In another study, various conjugative pVir plasmids containing type IV secretion system genes and other virulence genes have been described and were shown to be important for pathogenesis [67]. Mutations in pVir-associated type IV secretion system genes, for instance, caused a reduction in the ability of *C. jejuni* to attach to and invade intestinal epithelial cells *in vitro* and cause disease in ferrets [57,67]. Although we extracted the plasmid sequences from genomic versus plasmid DNA prepared with the Illumina Nextera XT kit for short-read sequencing, which could have resulted in uneven coverage [68] and an underestimate of plasmid diversity, we were still able to detect three distinct pVir-like plasmids with varying distributions using MOB-suite v.3.1.9 [39]. These data demonstrate that a diverse group of conjugative virulence plasmids is circulating within cattle- and human-derived *C. jejuni* populations in Michigan, with some belonging to specific CCs and clusters in the core-gene phylogeny (Fig. 6). For instance, the pFORC46.2 plasmid, originally isolated from a campylobacteriosis case in Korea, was most common in the human- and cattle-derived strains within the ST-982 (CC-21) and CC-61 cattle specialist lineages. Upon further analysis, we found one pFORC46.2 plasmid from a human-derived ST-982 strain, which has features associated with conjugative transfer [58], to be highly similar to the pFORC46.2 plasmids from a related human- and cow-derived CC-61 strain (Fig. 7). This finding suggests that these plasmids move freely between related strains and get propagated through progeny, which is important for adaptation to new hosts and conditions, as was suggested previously [69]. Additional studies, however, are needed to determine how these plasmids contribute to *C. jejuni* fitness, survival and pathogenesis in humans.

It is notable that the core-genome phylogenetic reconstruction demonstrates intermixing of cattle- and human-derived strains, particularly within the ST-982 and CC-61 clusters (Fig. 4). The high degree of genomic similarity between strains from both sources is consistent with our prior study showing highly similar rep-PCR fingerprint profiles among a subset of the cattle and human ST-982 strains [18]. Nonetheless, our study is limited in that we do not know the actual source of each human strain examined in the comparative analysis. Even though our prior study of these human strains identified an association between ST-982 and cattle contact [19] and the aiSource machine learning tool predicted a cattle origin for all ST-982 strains [21], prior case investigations did not uncover the source. This is not surprising because the majority of human infections are sporadic, yet a wide range of contaminated foods and sources have been reported [5]. To understand transmission dynamics in cows and humans, longitudinal cohort studies are necessary, while case investigations require confirmation of an identical strain from a suspected source using WGS [70]. In addition, our finding that the closest human-derived ST-982 ancestor differs from the cattle ST-982 strains by >260 SNPs (Fig. 5) provides additional evidence of diversification of this lineage within the cattle reservoir. Diversification of *C. jejuni* within cattle is consistent with a prior study showing that cattle-specific lineages (e.g. CC-61) emerged from generalist lineages in the 20^th^ century when the number of cattle increased globally [13]. Moreover, the cattle strains have less unique and accessory genes (Fig. 1) than the human strains as well as fewer virulence genes (Fig. 3a). Some of these genomic traits have been described previously and were suggested to be indicative of specialization and adaptation to cattle [13,61]. Since the cattle sampled in the study may not be representative and many of the unique genes encode for hypothetical proteins, more population-based studies are needed as well as use of updated annotation tools and databases in the future. Additional studies could also focus on understanding accessory gene and recombination networks among cattle-associated lineages to evaluate evolutionary pressures and identify genes under selection in this reservoir.

Although aiSource predicted all of the human-derived ST-982 *C. jejuni* strains to be cattle-associated (Fig. 4), the source of the three human-derived CC-61 strains was not cattle. Rather, these strains were predicted to originate from sheep and chickens despite clustering together with numerous cattle-derived strains from four different herds. This finding raises the possibility that some source predictions may be inaccurate. Since aiSource was trained on a global dataset of *C. jejuni* and *C. coli* genomes from mostly cattle and poultry [21], it is less useful for region- and lineage-specific datasets. Regardless, the identification of highly similar core genomes among cattle from different herds suggests that transmission of certain strain types occurs readily between reservoir hosts within and across farms. This suggestion is consistent with several studies of *C. jejuni* collected from multiple sources in different geographic locations, as similar strain types have been detected in cattle and chickens housed nearby [6], for example. Future studies, however, are needed to identify bacterial and host factors that facilitate *C. jejuni* survival in cattle and other reservoir species.

To further characterize strains belonging to the cattle-associated ST-982 lineage, we extracted 723 publicly available genomes representing ST-982 strains recovered from various sources in the USA. Using cgMLST, we observed clustering of the strains that was confirmed by a PCoA (Fig. 8a, b). Although sampling bias is a concern in population genomic studies, a particular strength of this analysis is the inclusion of all ST-982 genomes from PubMLST [34] (accessed January 2025). While most sequences deposited are from animal sources, namely cattle, seven represent our strains from humans with campylobacteriosis described here and in our prior study [27]. Fitting with the concept of a ‘geotype’, sequence clusters appear to be diversifying in a regionally or geographically specific environment [71]. In Michigan, the strains collected during the earliest years belong to cluster 1, whereas those recovered later were mostly part of cluster 2, which had significantly larger genomes. A cluster 3 bovine strain also emerged in Michigan in 2023. The same was true for other regions, which had varying cluster distributions with changes over time. Although no difference was observed across reservoir hosts by region, new hosts (e.g. goat, sheep and swine) emerged in various regions after 2018, which could be followed by a period of adaptation, diversification and spread among these hosts, as was demonstrated for cattle [13]. Because of unique selective pressures (e.g. agricultural practices, cattle densities and climate) in specific regions, the distribution of ST-982 strain types could continue to change with new clusters and reservoir hosts emerging. Since these *C. jejuni* strain types have the capacity to infect humans and cause campylobacteriosis, continuous monitoring is critical, as is defining the role that different reservoir hosts play in the maintenance and evolution of this important pathogen.

Altogether, our findings highlight how comparative genomics can be used to characterize strains circulating in both human and animal reservoirs to define similarities and differences that may make them more transmissible, host-restricted or virulent, for example. This analysis also illustrates the importance of the One Health approach, an integrated strategy for examining infectious disease agents in clinical, animal and environmental sources, which relies on local, regional and national surveillance efforts. Using genomics to enhance existing surveillance efforts will increase our ability to detect closely related strains across sources and locations as well as emergent lineages with unique traits. This approach can also help identify clinically important resistance determinants harboured by certain bacterial hosts, which may be more common in some environmental or animal reservoirs due to specific selective pressures. When combined with epidemiological data, these efforts can guide site-specific mitigation strategies for foodborne pathogens with complex lifestyles like *C. jejuni* and lead to real-time inferences about transmission and source attribution that can inform public health action.

## Supplementary material

10.1099/mgen.0.001553 Uncited Supplementary Material 1.

## Abbreviations

BIGSdb: Bacterial Isolate Genome Sequence Database
CC: clonal complex
cgMLST: core-gene MLST
CI: confidence interval
hqSNP: high-quality SNP
iTOL: Interactive Tree Of Life
MDR: multidrug resistance
MLK: macrolide, lincosamide and ketolide
MLST: multilocus sequence typing
NCBI: National Center for Biotechnology Information
OR: odds ratio
PcoA: principal coordinate analysis
PERMANOVA: permutational multivariate ANOVA
rep-PCR: repetitive element sequence-based PCR
SET: SNP extraction tool
ST: sequence type
VFDB: virulence factor database
WGS: whole-genome sequencing
